# Robust Classification and Detection of Big Medical Data Using Advanced Parallel *K*-Means Clustering, YOLOv4, and Logistic Regression

**DOI:** 10.3390/life13030691

**Published:** 2023-03-03

**Authors:** Fouad H. Awad, Murtadha M. Hamad, Laith Alzubaidi

**Affiliations:** 1 College of Computer Science and Information Technology, University of Anbar, Ramadi 31001, Iraq; 2 Faculty of Science and Engineering, Queensland University of Technology, Brisbane, QLD 4000, Australia; 3 ARC Industrial Transformation Training Centre-Joint Biomechanics, Queensland University of Technology, Brisbane, QLD 4000, Australia; 4 Centre for Data Science, Queensland University of Technology, Brisbane, QLD 4000, Australia

**Keywords:** medical data, medical imaging, data classification, image detection, YOLOv4, logistic regression, machine learning, AI, deep learning

## Abstract

Big-medical-data classification and image detection are crucial tasks in the field of healthcare, as they can assist with diagnosis, treatment planning, and disease monitoring. Logistic regression and YOLOv4 are popular algorithms that can be used for these tasks. However, these techniques have limitations and performance issue with big medical data. In this study, we presented a robust approach for big-medical-data classification and image detection using logistic regression and YOLOv4, respectively. To improve the performance of these algorithms, we proposed the use of advanced parallel *k*-means pre-processing, a clustering technique that identified patterns and structures in the data. Additionally, we leveraged the acceleration capabilities of a neural engine processor to further enhance the speed and efficiency of our approach. We evaluated our approach on several large medical datasets and showed that it could accurately classify large amounts of medical data and detect medical images. Our results demonstrated that the combination of advanced parallel *k*-means pre-processing, and the neural engine processor resulted in a significant improvement in the performance of logistic regression and YOLOv4, making them more reliable for use in medical applications. This new approach offers a promising solution for medical data classification and image detection and may have significant implications for the field of healthcare.

## 1. Introduction

The advancement of digital medical technology, coupled with the exponential growth of medical data, has led to biomedical research becoming a data-intensive science, resulting in the emergence of the “big-data” phenomenon, as reported in the literature, such as in [1]. Data have become a strategic resource and a key driver of innovation in the era of big data, transforming not only the way biomedical research has been conducted, but also the ways in which people live and think, which has been highlighted in studies such as [2]. To capitalize on this, the relevant departments in the medical industry should focus on collecting and managing medical health data and use this information as a foundation for later developments through the integration, analysis, and application requirements required to employ big data in the medical field [3].

Big medical data and image detection is an essential element of healthcare that plays a critical role in the storage, organization, and analysis of medical information [4]. the effective classification of medical data enables the efficient retrieval and examination of patient records, which can aid in the diagnosis and treatment of illnesses. It can also assist in identifying trends and patterns in patient health data, enabling healthcare professionals to recognize potential risk factors and take preventative measures. Furthermore, medical data classification has facilitated the advancement of new treatments and therapies by allowing researchers to analyze large datasets and uncover potential correlations and trends [5].

COVID-19 data classification has involved organizing and labeling data related to the coronavirus pandemic, such as information about confirmed cases, deaths, and vaccination rates. These types of data have often been used to track the spread of the virus and inform public health decisions. Image detection techniques have been used to identify COVID-19-related images, such as X-ray scans showing lung abnormalities associated with the virus. These techniques have assisted healthcare professionals and researchers better understand and track the spread of the virus.

However, there have been several challenges and problems associated with COVID-19 data classification and image detection. One major challenge has been ensuring the accuracy and reliability of the data being used. There have been errors and biases in the data that affected the results. Additionally, there have been privacy concerns related to collecting and using personal health data. There have also been technical challenges in developing and implementing image detection algorithms, such as difficulties in obtaining a sufficiently large dataset for training. Overall, addressing these challenges is crucial in order to effectively use data and image detection techniques to understand and combat the COVID-19 pandemic.

In this study, an efficient and high-performance solution to enhance the accuracy of medical data classification and image detection was proposed. Advanced *k*-means clustering was merged with both classification and detection techniques to elevate the performance and accuracy of these techniques [6]. To evaluate the performance of medical data classification, a large medical dataset was used. Furthermore, to evaluate the effectiveness of the detection technique, a dataset comprising X-ray COVID-19 and CT images was utilized. The results indicated that the proposed models significantly improved the performances of classification and detection. The proposed model’s contributions were the following:

1. The successful application of advanced parallel *k*-means clustering as a pre-processing step for both the images and the data to improve the accuracy of image feature extraction and detection, as well as the accuracy of data classification.
2. Both hardware and software improvements were employed to significantly accelerate the classification and detection processes. Hardware acceleration was achieved by utilizing the latest neural engine processor while the software optimization involved using parallel-processing mechanisms.

This paper is divided into seven sections. The introduction addresses the significance of medical data classification and medical image detection. Section 2 discusses various data classification and image detection algorithms, including their advantages and limitations. Section 3 addresses the current challenges and features of solutions for processing large amounts of medical data and images. Section 4 presents the proposed solution. Section 5 outlines the methodology and performance metrics used to evaluate the proposed solution. The implementation and results of the proposed solution are presented in Section 6. Section 7 concludes the paper.

## 2. Data Classification

Data classification is the process of organizing and categorizing data based on predetermined criteria [7]. It is a crucial aspect of many applications, including data management, data analysis, and information retrieval.

### Logistic Regression Algorithm

Logistic regression is a type of binary classification algorithm that is used to predict the probability of an event occurring [8]. It has been commonly used in machine learning for applications such as spam detection, medical diagnosis, and sentiment analysis [9].

The logistic-regression model maps the input features $x_{1},x_{2},...,x_{n}$ to a predicted output variable *y* that has a value between 0 and 1, representing the probability of the event occurring [10].

The logistic function, also known as the sigmoid function, is used to model the relationship between the input features and the predicted output variable. The sigmoid function is defined as [10]:

$$f\left(z\right)=\frac{1}{1+e^{-z}}$$

where $z=w_{0}+w_{1}x_{1}+w_{2}x_{2}+...+w_{n}x_{n}$ is the linear combination of the input features and their corresponding weights, with $w_{0}$ as the bias term.

The logistic regression algorithm aims to find the optimal values for the weights $w_{0},w_{1},w_{2},...,w_{n}$ that minimize the error between the predicted output variable and the true output variable [11]. This is achieved by maximizing the likelihood function, which is the probability of the observed data according to the model parameters [12]. The likelihood function for logistic regression is:

$$L\left(w\right)=\prod_{i=1}^{m}f{\left(z_{i}\right)}^{y_{i}}{\left(1-f\left(z_{i}\right)\right)}^{1-y_{i}}$$

where *m* is the number of training examples, $y_{i}$ is the true output variable for the *i*th example, and $z_{i}$ is the linear combination of the input features and weights for the *i*th example [13].

The optimal values for the weights can be found using gradient descent, which involves iteratively updating the weights in the direction of the negative gradient of the likelihood function [14]. The updated rule for the weights is:

$$w_{j}:=w_{j}-\alpha \frac{\partial L(w)}{\partial w_{j}}$$

where α is the learning rate, and $\frac{\partial L(w)}{\partial w_{j}}$ is the partial derivative of the likelihood function with respect to the *j*th weight.

The logistic regression algorithm can be summarized in the following steps:

1. Initialize the weights $w_{0},w_{1},w_{2},...,w_{n}$ to random values.
2. Calculate the linear combination $z_{i}$ for each training example using the current weights.
3. Calculate the predicted output variable $y_{i}$ for each training example using the logistic function.
4. Calculate the error between the predicted output variable and the true output variable for each training example.
5. Calculate the gradient of the likelihood function with respect to each weight.
6. Update the weights using the gradient descent update rule.
7. Repeat steps 2–6 until the error converges or a maximum number of iterations is reached.

One of the main advantages of logistic regression is its simplicity and ease of implementation. It is a straightforward algorithm that can be easily implemented using standard statistical software [15]. Additionally, logistic regression is highly interpretable, allowing users to understand the contributions of each independent variable to the predicted probability. It is also robust regarding multicollinearity, meaning that it can handle correlated independent variables without producing biased estimates.

However, logistic regression is not without its challenges. One of the main limitations is that it is only suitable for binary classification problems, meaning that it can only predict the likelihood of an event occurring or not occurring [16].

## 3. Image Detection Technique

Image detection is a technique used to identify and locate specific objects, features, or patterns within an image. It is a crucial aspect of many applications, including object recognition, facial recognition, and scene comprehension [17]. In the field of healthcare, image detection is used to analyze and interpret medical images, such as X-rays, CT scans, and MRIs. These images provide important diagnostic information that can be used to identify and treat diseases.

### YOLOv4 Algorithm

The You Only Look Once version 4 (YOLOv4) algorithm is a state-of-the-art object detection algorithm that processes an entire image and directly predicts the bounding boxes and class probabilities for all objects in the image. It uses a convolutional neural network (CNN) to extract features from the input image and then apply them a series of convolutional and fully connected layers in order to predict the class probabilities and bounding box coordinates for each object [18].

The YOLOv4 algorithm predicts object classes and bounding box coordinates by dividing the input image into a grid of cells and predicting the class probabilities and bounding box offsets for each cell [19]. Specifically, for each cell in the grid, the algorithm predicts:

- The probability of an object being present in that cell (denoted $p_{obj}$).
- The x and y coordinates of the center of the bounding box, relative to the coordinates of the cell (denoted by $b_{x}$ and $b_{y}$, respectively).
- The width and height of the bounding box relative to the size of the cell (denoted by $b_{w}$ and $b_{h}$, respectively).
- The class probabilities for each object class (denoted by $p_{c1}$, $p_{c2}$,…, $p_{cn}$, where *n* is the number of classes).

These predictions are made using a series of convolutional and fully connected layers in the YOLOv4 network. The network architecture is based on a variant of the DarkNet architecture, which consists of multiple convolutional layers and followed by max-pooling layers, and ends with multiple fully connected layers [20]. The final layer of the network outputs a tensor that is the shape of (grid size) × (grid size) × (number of anchor boxes) × (5 + number of classes), where the 5 refers to the objectness score, $b_{x}$, $b_{y}$, $b_{w}$, and $b_{h}$ [21].

The YOLOv4 algorithm then uses non-maximum suppression to remove redundant bounding boxes for the same object [22]. Specifically, for each class, it applies non-maximum suppression to the set of predicted bounding boxes with objectness scores above a certain threshold. This threshold is usually set to a value between 0.5 and 0.7, depending on the desired balance between precision and recall [23].

The YOLOv4 algorithm can be trained using a loss function that measures the errors between the predicted and ground-truth bounding boxes and class probabilities [24]. The loss function consists of two components: a localization loss that penalizes errors in the predicted bounding box coordinates, and a classification loss that penalizes errors in the predicted class probabilities. The localization loss is typically computed using the mean squared error (MSE) between the predicted and ground-truth bounding box coordinates, while the classification loss is typically computed using the cross-entropy loss between the predicted and ground-truth class probabilities [25].

Algorithm 1 shows the main steps of the YOLOv4 algorithm.
**Algorithm 1** YOLOv4 object detection algorithm**Require:** Input image *I* **Ensure:** Bounding boxes *B* and class probabilities *C*
1:Pre-process *I* to obtain an input tensor *X*2:Apply the backbone network to obtain feature maps $F_{1},F_{2},...,F_{n}$3:Apply the neck network to combine the feature maps and obtain a single feature map *F*4:Apply the detection head to *F* to obtain a set of candidate boxes $B_{c}$ and class probabilities $C_{c}$5:Apply non-maximum suppression (NMS) to $B_{c}$ and $C_{c}$ to obtain the final set of bounding boxes *B* and class probabilities *C*, respectively6:**return** *B* and *C*


Note that this algorithm assumes that the YOLOv4 architecture has already been trained on a large dataset of images with labeled objects and that the resulting model has been saved and can be loaded for inferences on new images. The backbone network, neck network, and detection head are all components of the YOLOv4 architecture, and their specific details are beyond the scope of this pseudo-coded algorithm [26].

One way that YOLOv4 has been used in medical image analysis has been in the detection of abnormalities and lesions in images [27]. For example, it was used to identify abnormalities in CT scans of the brain, which could then be used to diagnose and treat brain tumors. By analyzing CT scans with YOLOv4, healthcare professionals could more accurately identify abnormalities and determine the appropriate course of treatment.

During the COVID-19 pandemic, YOLOv4 has also been used to analyze chest X-rays, which have often been used to diagnose the virus [28]. By detecting characteristic patterns associated with COVID-19, such as lung abnormalities, YOLOv4 assisted healthcare professionals in making accurate diagnoses and providing timely treatments for patients [29]. In addition to its use in detecting abnormalities within images, YOLOv4 has also been used to detect objects in images, such as medical instruments and organs. This was particularly useful for identifying and tracking objects during surgical procedures, such as in the detection of brain tumors [30].

## 4. Medical Data Classification and Detection

Medical data classification and image detection are two critical areas in healthcare that could benefit from the latest advancements in machine-learning and computer-vision technologies. In recent years, there has been a significant increase in the amount of medical data generated due to the availability of electronic health records and medical imaging technologies. This growth in medical data has provided new opportunities for developing more accurate and efficient methods for classification and image detection, which could lead to improved diagnoses, treatments, and patient outcomes.

### 4.1. Medical Data and Image Classification

The classification of medical data refers to the process of assigning a label or category to a particular medical dataset. The classification of medical data could be used for various applications, such as disease diagnosis, drug discovery, and prognosis. The following are the state-of-the-art techniques used in medical data classification.

- Deep Learning: Deep learning has revolutionized the field of medical data classification and image detection, due to its ability to handle large and complex datasets with improved accuracy and efficiency [31]. Convolutional neural networks (CNNs) and recurrent neural networks (RNNs) are two widely used deep-learning techniques that have demonstrated exceptional performance in the medical field [32]. CNNs have been specifically designed to analyze visual imagery, making them a popular choice for medical image analysis. They consist of multiple layers that learn different features of an image, such as edges and textures, and then use these features to classify the image. The ability of CNNs to automatically extract relevant features from medical images has led to their use in a wide range of applications, such as mammogram analysis for breast cancer detection and brain tumor segmentation. Furthermore, RNNs have been designed to process sequential data and have been extensively used in various medical applications, such as medical signal processing, clinical event prediction, and ECG signal analysis [33]. They are able to analyze the temporal dependencies in sequential data by using a memory component that allows them to remember past inputs and use them to influence future predictions. RNNs have also been used in combination with CNNs to analyze both image and sequential data, such as in the case of electroencephalogram (EEG) signal analysis [34]. In addition to CNNs and RNNs, other deep-learning techniques, such as generative adversarial networks (GANs) and auto-encoders have also been explored in medical data classification and image detection [35]. GANs have been used to generate synthetic medical images, which were then used to augment existing datasets and improve the performance of image classifiers. Auto-encoders, in contrast, have been used for feature extraction and dimensionality reduction, which improved the efficiency of classification algorithms.
- Support Vector Machines (SVMs): SVMs are a type of supervised learning algorithm that has been widely used for classification tasks in many areas, including in medical data classification. SVMs have been particularly useful for classification tasks in which the number of features was much greater than the number of samples [36]. SVMs find the optimal hyperplane that separates the different classes in a dataset. For medical data classification, SVMs have been used for tasks such as disease diagnosis, the classification of different types of cancer, and the identification of abnormal medical images [37]. SVMs have shown high accuracy and robustness in these tasks due to their ability to handle non-linear data and their resistance to over-fitting.
  One example of SVMs being used in medical data classification was for the identification of breast cancer using mammograms [38]. SVMs had a high accuracy in distinguishing between benign and malignant tumors, which is critical for the early detection and treatment of breast cancer. SVMs have also been used for the classification of brain tumors and the identification of Alzheimer’s disease in medical imaging data.
- Random Forest: Random forest is a type of ensemble learning algorithm that combines multiple decision trees to improve its classification accuracy. The method is considered a supervised learning technique that operates by constructing several decision trees during training and then predicts the class label of an input data point by aggregating the predictions of all the decision trees [39]. Random forest has been effective in medical data classification due to its ability to handle high-dimensional data and its resistance to over-fitting. In medical applications, random forest has been used for various classification tasks, such as disease diagnosis, the prediction of treatment responses, and mortality risk assessments [40]. One advantage of random forest is its ability to handle missing data and noisy features. This is achieved by randomly selecting a subset of features at each node in the decision tree, which reduces the risk of over-fitting and improves the model’s generalization performance. Additionally, the method allows for the calculation of feature importance, which can help identify the most important variables that contribute to the classification task.

However, the classification of medical data has not been without challenges. One of the main challenges has been the large volume of data that must be classified [41]. Medical data are typically generated at a rapid rate and can be difficult to manage due to size and complexity. Additionally, medical data classification often involves working with sensitive and personal information, which requires strict adherence to the privacy and security measures in place. Another challenge has been the lack of standardization in medical data classification, which has led to confusion and difficulties in data retrieval and analysis [42]. Finally, the constantly evolving nature of the healthcare field means that medical data classification systems must be regularly updated and adapted to meet changing needs.

### 4.2. Medical Image Detection

Image detection in healthcare refers to the process of detecting and identifying medical conditions or abnormalities in medical images such as X-rays, CT scans, and MRI scans. Image detection plays a vital role in the diagnosis and treatment of various medical conditions [43]. The following are the state-of-the-art techniques used in image detection in healthcare.

- Convolutional Neural Networks (CNNs): In medical imaging, CNNs have been used for a variety of applications, such as the detection of breast cancer, lung cancer, and brain tumors [44]. For example, in breast cancer detection, CNNs have been used to analyze mammograms and detect subtle changes that could indicate the presence of cancer. In lung cancer detection, CNNs have been used to analyze CT scans and identify nodules that could be indicative of cancer. In brain tumor detection, CNNs have been used to analyze MRI scans and identify regions of abnormal tissue growth [45].
  One of the advantages of using CNNs for medical image detection is their ability to learn and extract features automatically, without the need for manual feature extraction [46]. This makes them particularly useful for analyzing large and complex medical images, where manual feature extraction can be time-consuming and prone to error. Another advantage of CNNs is their ability to learn from large amounts of data. With the increasing availability of medical imaging data, CNNs can be trained on large datasets to improve their accuracy and generalization performance [47]. Additionally, CNNs can be fine-tuned and adapted for specific medical image detection tasks, which can further improve their performance.
- Transfer Learning: In the context of medical image detection, transfer learning was an effective method for improving the accuracy and efficiency of image classification tasks [48]. Pre-trained models, such as those based on CNNs, can learn generic image features that can be transferred to new medical imaging datasets, even when the size of the new dataset is relatively small [49]. This can be particularly useful in healthcare, where obtaining large labeled datasets can be challenging and time-consuming. By using transfer learning, researchers and clinicians leveraged the knowledge and expertise gained from pre-trained models to improve the accuracy and efficiency of image detection in healthcare [50]. For example, a pre-trained model that was trained on a large dataset of chest X-rays was then fine-tuned for a smaller dataset of lung cancer images, resulting in improved accuracy and faster training times.

## 5. Related Works

A literature review was conducted to examine the most recent approaches and techniques for medical data classification in this field.

The related works presented here were selected based on their technological similarity to the proposed solution and their focus on medical data. Furthermore, all papers were chosen based on their publication in high-quality journals. Furthermore, as COVID-19 has attracted the attention of researchers in the healthcare field, most of the papers selected in this review were related to the global COVID-19 pandemic.

In [51], the aim was to evaluate the performance of parallel computing and advanced *k*-means clustering as a pre-processing step for data classification and image detection in medical applications. To achieve this, the researchers utilized a parallel logistic regression algorithm and a mobile neural engine processor. The *k*-means clustering technique was used to pre-process both images and data, resulting in improvements in feature extraction, the removal of noise and outlier pixels, and classification accuracy. The results of this study showed that their proposed approach outperformed traditional methods both in terms of both accuracy and efficiency, making it a promising approach for medical data analysis and processing.

In 2021, the researchers in [52] proposed a new method for optimizing the performance of the *k*-means clustering algorithm on parallel and distributed computing systems. The study employed a hybrid approach that combined the traditional Lloyd’s algorithm with a new partitioning technique. The proposed approach was evaluated using various datasets, and the results showed that the hybrid approach outperformed both the traditional Lloyd’s algorithm and other state-of-the-art parallel *k*-means algorithms, in terms of both accuracy and efficiency. The study concluded that the proposed approach was a promising solution for large-scale clustering tasks on parallel and distributed computing systems. In [53], the authors proposed a new framework for automating the diagnosis of Alzheimer’s disease (AD) using a machine-learning approach. The proposed framework utilized a combination of several machine-learning algorithms, including principal component analysis (PCA), support vector machine (SVM), and *k*-nearest neighbors (KNN) to classify brain images as normal or AD. The study used two different datasets, and the results showed that the proposed framework achieved high accuracy and specificity when classifying brain images as AD. The study concluded that the proposed framework could be a valuable tool for the early diagnosis and monitoring of AD.

In 2022, [54] investigated the potential use of deep learning algorithms for the detection of COVID-19 in chest X-ray images. The study proposed a deep-learning model based on convolutional neural networks (CNNs) that had been trained on a large dataset of chest X-ray images. The model was tested on a separate dataset of chest X-ray images, and the results showed that the proposed model achieved high accuracy, sensitivity, and specificity, in detecting COVID-19. The study concluded that the proposed deep-learning model could be a valuable tool for the rapid and accurate detection of COVID-19 in chest X-ray images, especially in regions with limited access to COVID-19 testing facilities.

A literature review was conducted in order to review the most recent approaches and techniques for medical image detection.

The authors of [55] developed a machine-learning algorithm that could accurately classify patients with severe COVID-19 and predict their risk of in-hospital mortality. The study collected data from electronic health records of patients with severe COVID-19, including demographics, vital signs, laboratory values, and comorbidities. A machine-learning algorithm based on a gradient-boosting machine (GBM) was developed and trained on the collected data. The results showed that the proposed GBM model achieved high accuracy in classifying patients with severe COVID-19 and predicting their risk of in-hospital mortality. The study concluded that the proposed machine-learning algorithm could be a valuable tool for clinicians to make more informed decisions about the management of patients with severe COVID-19.

In 2021, the authors of [56] proposed a classification solution using transfer learning to assess the suitability of 3 pre-trained CNN models (EfficientNetB0, VGG16, and InceptionV3) for mobile applications. These models were selected for their accuracy and efficiency with a relatively small number of parameters. The study used a dataset compiled from various publicly available sources and evaluated the models using performance measurements and deep-learning approaches, such as accuracy, recall, specificity, precision, and F1-scores. The results demonstrated that the proposed method produced a high-quality model with a COVID-19 sensitivity of 94.79% and an overall accuracy of 92.93%. The study suggested that computer-vision techniques could be utilized to improve the efficiency of detection and screening processes.

In 2021, the authors of [57] employed convolutional neural networks (ConvNets) to accurately identify COVID-19 in computed tomography (CT) images, enabling the early classification of chest CT images of COVID-19 by hospital staff. ConvNets automatically learned and extracted features from medical image datasets, including the COVID-CT dataset used in this study. The objective was to train the GoogleNet ConvNet architecture using 425 CT-coronavirus images from the COVID-CT dataset. The experimental results indicated that GoogleNet achieved a validation accuracy of 82.14% on the dataset in 74 min and 37 s. This study demonstrated the potential of ConvNets in improving the accuracy and efficiency of COVID-19 detection in medical imaging.

In 2022, the authors of [58] proposed a new method for improving the quality of CT scans using contrast limited histogram equalization (CLAHE) and developed a convolutional neural network (CNN) model to extract important features from a dataset of 2482 CT-scan images. These features were then used as input for machine-learning methods such as support vector machine (SVM), Gaussian naive Bayes (GNB), logistic regression (LR), random forest (RF), and decision tree (DT). The researchers recommended an ensemble method for classifying COVID-19 CT images and compared the performance of their model with other state-of-the-art methods. The proposed model outperformed existing models with an accuracy of 99.73%, a precision of 99.46%, and a recall of 100%.

In 2022, the authors of [59] described an approach that used a generative adversarial network (GAN) to improve the accuracy of a deep-learning model for classifying COVID-19 infections in chest X-ray images. To generate additional training data, the COVID-19 positive chest X-ray images were fed into a styleGAN2 model, which produced new images for training the deep-learning model. The resulting dataset was used to train a CNN binary classifier model that achieved a classification accuracy of 99.78%. This method could aid in the rapid and accurate diagnosis of COVID-19 infections from chest X-ray images.

## 6. Proposed Solution

The proposed solution was designed with two main objectives: medical data classification and medical image detection. Each model is described in detail in this section.

### 6.1. Advanced Parallel *K*-Means Clustering

In order to implement the modified parallel *k*-means clustering on the mobile execution unit and the SoC, the algorithm had to be modified to take advantage of a multi-core general-purpose processor and a multi-core neural engine. Each operating system offered a unique set of utilities for parallel operation.The iOS environment, due to its use of Objective-C programming, has an additional tool called dispatch queues, in addition to standard tools, such as processes and threads. Although iOS is a multi-tasking operating system, it did not allow multiple processes for a single program, resulting in only one procedure being available.

However, the Android OS had a limitation in its Java and Kotlin programming languages, which was the hardware-limited access and lack of pointer support, making it difficult to fully utilize the system hardware. A lightweight process is a thread of any type. Threads share memory with their parent process while processes themselves do not. This led to issues when two threads simultaneously modified the same resource, such as a variable, resulting in illogical outcomes. In the iOS environment, threads were a finite resource on any POSIX-compliant system. Only 64 threads could be active at once for a single process. While this is a large number, there were logical reasons to exceed this limit.

**Figure 1:**
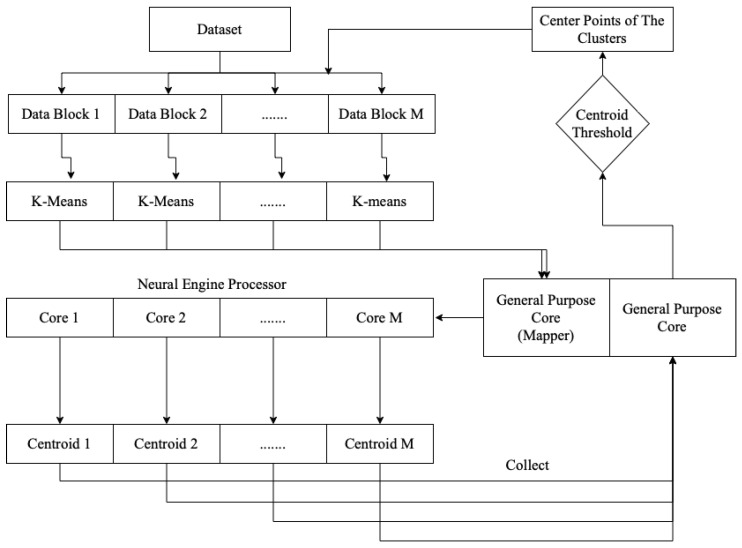
On-device parallel clustering processing.

The overall processing, as shown in Figure 1, of the on-device parallel clustering consisted of two jobs: managing the dataset and clustering execution, and performing the parallel *k*-means clustering itself. The general-purpose processor cores were responsible for managing the clustering in the neural engine cores. After executing the *k*-means clustering on a sub-block of the data, each core sent the centroid point-value to the general-purpose cores. The general-purpose cores then evaluated whether the centroid value was less than the centroid threshold. If it was less, a signal was sent to the execution mechanism to process the clustering again.

**Figure 2:**
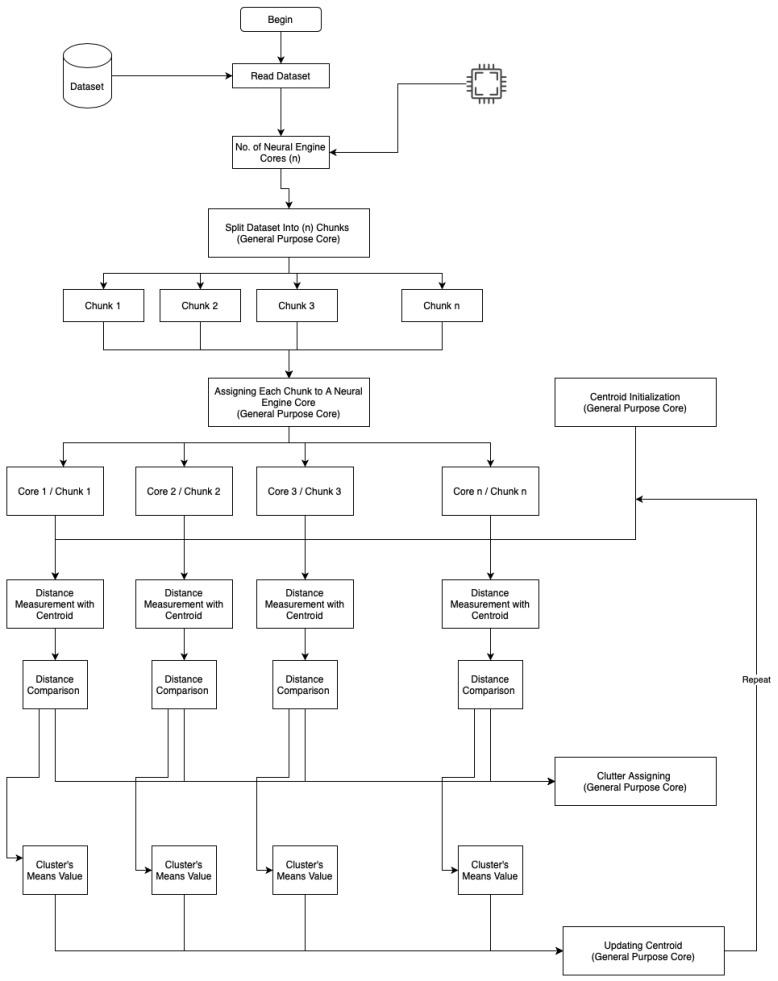
On-device parallel clustering flowchart.

Figure 2 shows a flowchart of advanced parallel *k*-means clustering on the neural engine and general-purpose cores.

### 6.2. Advanced Classification Solution

Pre-processing medical data with advanced parallel *k*-means clustering was a useful technique to improve the classification performance of logistic regression algorithms. *K*-means clustering is a machine-learning algorithm that is used to partition a dataset into a specified number of clusters. By using advanced parallel techniques, it is possible to process data more efficiently and quickly.

Pre-processing the medical data with *k*-means clustering improved the accuracy and precision of the logistic regression algorithms by ensuring the data were simpler to classify. The *k*-means algorithm divided the data into clusters based on similar characteristics, such as age or sex. This assisted in reducing the noise and the complexity of the data, making it simpler for the logistic regression algorithm to accurately classify the data.

In addition to improving the accuracy and precision of the classification process, pre-processing the medical data with *k*-means clustering also reduced the computational resources required to operate the logistic regression algorithm. By reducing the size and complexity of the dataset, it was possible to operate the logistic regression algorithm more efficiently and quickly.

After clustering the data using *k*-means clustering, the next step in the process was to perform the logistic-regression classification. The steps for performing parallel logistic-regression classification were the following:

- Pre-processing: As with non-parallel logistic regression, it was important to pre-process the data before applying the model. This included tasks such as missing-value imputation, scaling, and feature selection.
- Splitting the data: The data had to be split into training and testing sets in order to evaluate the model’s performance on unfamiliar data.
- Choosing a parallelization method: We had to decide whether to use data parallelism, model parallelism, or a hybrid parallelism.
- Partitioning the data: Depending on the chosen parallelization method, the data had to be partitioned into smaller chunks and distributed across multiple processors or devices.
- Training the model: Each processor or device was responsible for training a separate logistic-regression model on its chunk of the data. The models were then combined to form the final model.
- Evaluating the model: The trained model was then evaluated on the testing data. This involved calculating evaluation metrics, such as accuracy, precision, and recall.
- Assessing the model’s predictions: Once the model had been trained and evaluated, it was used to make predictions according to new data. To achieve this, the model’s parameters were used to calculate the probability of an instance belonging to each class. The class with the highest probability was then predicted as the output.

In Algorithm 2, the input is the data *D* and the number of processors or devices *n* to be used for parallelization. The output is the trained logistic-regression model *M*. The data were pre-processed and split into training and testing sets. The parallelization method was chosen, and the training data were then partitioned into smaller chunks. A separate logistic-regression model was trained on each chunk of data, and the models were combined to form the final model. The model was then evaluated on the testing data and the returned results.
**Algorithm 2** Parallel Logistic-Regression Classification1:**procedure** ParallelLogisticRegressionClassification(*D*, *n*)2:       Pre-process data *D*3:       Split data into training and testing sets $D_{train}$ and $D_{test}$4:       Partition data $D_{train}$ into *n* smaller chunks $D_{train,1},D_{train,2},\cdots ,D_{train,n}$5:       **for** i←1 **to** *n* **do**6:             Train logistic-regression model $M_{i}$ on chunk $D_{train,i}$7:       **end for**8:       Combine models $M_{1},M_{2},\cdots ,M_{n}$ to form final model *M*9:       Evaluate model *M* on testing data $D_{test}$10:     **return** Model *M*11:**end procedure**

The algorithm had two input parameters. The first was the clustered dataset, which included a new feature extracted by the clustering process. The second input was the number of chunks into which the dataset would be partitioned. The number of partitions depended on the number of neural engine cores available, with each chunk trained on a single core. The standard CPU cores handled general tasks, such as data partitioning; reading and writing data for the neural engine cores; combining models (M1, M2,…, Mn); and evaluating models.

#### Classification Pre-Processing

Using *k*-means clustering as a pre-processing step could potentially improve the performance of the logistic-regression classification in several ways:

- Dimensionality reduction: *K*-means clustering was used to group similar data points together into clusters, which reduced the number of features in the dataset. By selecting the centroids of the clusters as the new features, we reduced the dimensionality of the data and removed the noise, which improved the performance of the logistic regression.
- Feature engineering: *K*-means clustering was used to create new features that captured the structure of the data. We added a new binary feature that indicated whether a data point belonged to a particular cluster or not. These new features enabled the logistic regression to capture complex relationships in the data that had not been apparent previously.
- Outlier detection: *K*-means clustering improved the identification and removal of outliers in the dataset. Outliers had a significant impact on the performance of the logistic regression, and removing them improved the accuracy of the model.
- Data normalization: *K*-means clustering was used to normalize the data by scaling it to a range from 0 to 1. Normalizing the data improved the performance of the logistic regression by reducing the impact of outliers and ensuring that all features were on a similar scale.

In the proposed parallel logistic regression, the weighted-combination method assisted in forming the final logistic-regression model from individual models that had been trained by each processor or device. An overview of the process is provided:

- Train individual models: The dataset was divided into subsets, and each subset was used to train a logistic-regression model on a separate processor or device.
- Obtain model weights: Once the individual models had been trained, each model was assigned a weight based on its performance on a validation set. The weights were determined using a variety of methods, such as the accuracy or the area under the receiver-operating characteristic curve (AUC-ROC).
- Combine the models: The predicted probabilities or coefficients from each individual model were multiplied by their corresponding weights, and the weighted sum was used as the final output. For example, if there were three individual models with weights of 0.3, 0.5, and 0.2, the predicted probabilities of each model were multiplied by 0.3, 0.5, and 0.2, respectively, and then summed to obtain their final predicted probabilities.
- Model selection: The performance of the final model was evaluated on a validation set, and the weights assigned to the individual models were adjusted to improve the performance of the final model. This process was repeated until the desired level of performance was achieved.
- Apply the final model: Once the final model was selected, it was implemented to make predictions on new data.

The weighted-combination method can be an effective way to leverage the power of multiple processors or devices to train logistic-regression models in parallel. By assigning weights to each individual model, the final model can benefit from the strengths of each model while mitigating their weaknesses.

### 6.3. Advanced Image Detection

In this study, we proposed a novel approach for pre-processing images using advanced parallel *k*-means clustering and then applying image detection using YOLOv4. The *k*-means clustering algorithm was used to divide the images into segments, which were then processed in parallel by multiple processors. The parallel-processing of the image segments resulted in a significant reduction in the overall processing time. The *k*-means algorithm is a popular method for clustering data based on similarity. It groups similar data points together and forms clusters. In the proposed approach, *k*-means was used to divide the images into segments, where each segment represented a cluster of similar pixels. The parallel-processing of these segments was achieved by distributing the segments across multiple processors. This allowed for a more efficient use of resources and resulted in a significant reduction in the overall processing time.

After the image had been segmented, the image detection algorithm YOLOv4 was applied to each segment. YOLOv4 is a state-of-the-art object detection algorithm that has been widely used for image-processing tasks. It can accurately detect and classify objects in an image, making it an ideal choice for this application. The proposed approach provided several advantages over traditional image-processing methods. The use of advanced parallel *k*-means clustering allowed for a more efficient use of resources, resulting in faster processing times. Additionally, the application of YOLOv4 to the image segments improved the accuracy of object detection. Overall, the proposed approach was a powerful tool for image processing on mobile devices.

#### Stage 1: Image Clustering and Pre-Processing

The intricate structure of the information in images makes the clustering of X-ray (radiographs) and CT-scan images challenging. A considerable visual resemblance exists between X-ray and CT images of the same class. Furthermore, because of the varied X-ray image types, orientation changes, alignments, and diseases, there was a significant variance within a class. The quality of the X-ray images also varied significantly, in addition to the contents. As illustrated in the accompanying diagram, the image clustering framework in this study was divided into two phases: image feature extraction and image clustering.

Then, the clustering process was carried out using the machine-learning engine-specific processors in contemporary mobile devices. Maintaining dataset characteristics while improving clustering efficiency was recommended [6].

Algorithm 3 outlined the primary steps for clustering the pixels in the input image, using the modified *k*-means clustering algorithm, as described earlier in this section.
**Algorithm 3** *K*-Means Image Clustering**Require:** Image Dataset
     **Input:** Random Centroid Points
     **Start:** Clustering Pixels
     **while** pixels≠end **do**
           **Select:** Neural Engine Core
           **Assign:** Processing to Core
           **Calculate:** Mean Value
           **Set**: Pixel-to-Cluster
     **end while**
     **Output:** Clustered Pixels


Initially, patient X-ray and CT-scan images of COVID-19 disease were segmented using the *k*-means clustering algorithm, which then split the image into a set of regions that could be processed and analyzed. Due to the high performance achieved through the modification of the aforementioned algorithm, this step resulted in a thorough scan of the images and the segmentation of their content at a high speed, in preparation for the next stage, which was the application of the YOLOv4 algorithm.

Second, incoming images were resized to 640 by 640 px and normalized using a normalize procedure. The improved *K*-means clustering algorithm, based on mobile neural engine processors [6], was then used to further match the training data with the *k*-mean YOLOv4 model. A suitable anchor size setting facilitated model convergence and provided useful prior information, and this sped up the model training process and resulted in more accurate values. The full implementation flowchart of anchor sizes is provided.

**Figure 3:**
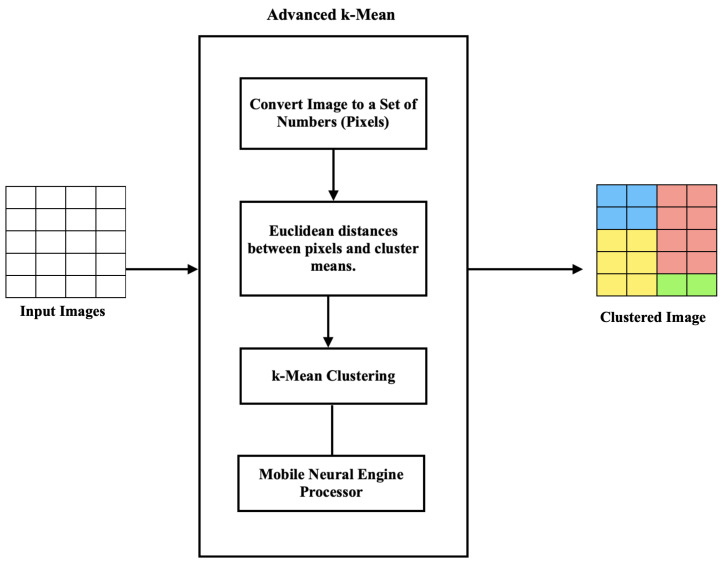
Stage 1 image clustering architecture.

Figure 3 summarizes the main steps of the first stage of image clustering.

By clustering pixels in an image, we simplified the image by reducing the number of colors and tones. This assisted in removing noise and unwanted details from the image, making it easier to extract relevant features.

Once the pixels were clustered, a new image was created where each pixel was assigned to its corresponding cluster, based on the map image. This new image was called a clustered image. The clustered image contained fewer colors and tones than the original image and could be used to extract features that were more representative of the image content.

For example, in the medical image analysis, *k*-means clustering was used to segment an X-ray or CT-scan image into regions based on the density of the tissue. By clustering the pixels in the image, we identified regions that corresponded to bones, organs, and other tissues, which were then evaluated for feature extraction. These features included the size, shape, and texture of the tissue, which was then used to detect abnormalities and other features that could be indicative of a disease or condition.

### 6.4. Stage 2: YOLOv4 Image Detection

**Figure 4:**
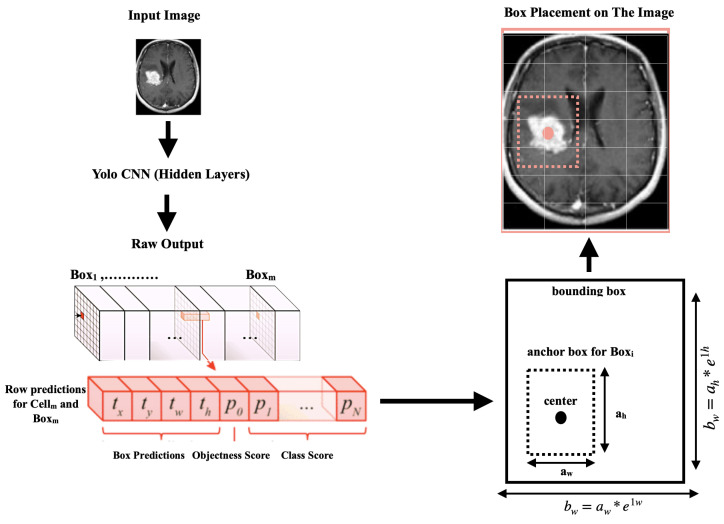
YOLOv4 image detection architecture.

After processing the images, the second stage of scanning the images commenced using the YOLOv4 algorithm, which could handle and detect objects in images at high speeds. Objects were easier to identify and detect in the pre-processed images due to the image content being segmented into consistent data aggregates.As shown in Figure 4, every object detector began by compressing and processing the images using a convolutional neural network backbone, which could then be used to make predictions at the endpoint of the image classification. To detect objects, several bounding boxes had to be constructed around images, requiring the concatenation of the convolutional feature layers of the backbone and the convergence of all the layers of features in the backbone at the neck.

The YOLOv4 system utilized image-resizing, non-maximal suppression, and a single convolutional neural network to identify objects. It generated multiple bounding boxes and class probabilities simultaneously. Although the system was efficient for detecting objects, it could have difficulty identifying the locations of smaller objects precisely.

The input images were divided into an S × S grid, with each grid cell responsible for identifying an object if the centroid of the object was within that grid cell. Using information from the entire image, each grid cell predicted the bounding boxes (B) and the confidence ratings for those boxes. These confidence scores represented the likelihood that an object was present in the box, as well as the accuracy of the object class prediction. The confidence score was defined as:

(1) $$conf=Pr\left(class_{i}|obj\right)\times Pr\left(obj\right)\times IoU_{pred}^{truth}$$

where

(2) Pre(obj)∈[0,1]

here, *Pr(object)* denotes the likelihood that there will be an object in the grid cell, and *Pr(classic|obj)* denotes the likelihood that a particular object will appear based on the presence of an item in the cell.

### 6.5. Stage 3: *K*-Means–YOLOv4 Clustering

**Figure 5:**
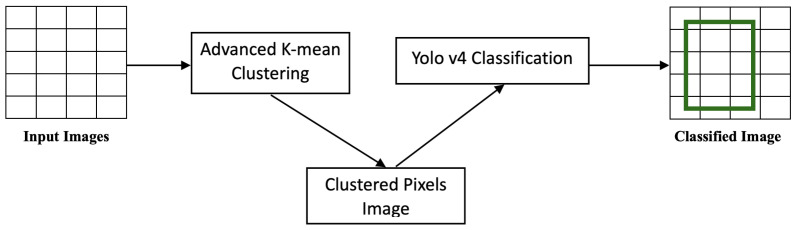
Proposed solution architecture.

YOLOv4 used Bag of Specials, which is a technique that adds minimal delays to inference times while significantly enhancing performance. The algorithm evaluated various activation functions. As features flowed through the network, the activation functions were altered, as depicted in Figure 5. Using conventional activation functions, such as ReLU, had not always been sufficient to push feature creation to its optimal limit, which has led to the development of novel techniques in the literature to slightly improve this method.

To summarize Stage 3 as an algorithm, Algorithm 4 was written. As shown in the algorithm, the YOLOv4 detector received the clustered image before initializing the YOLOv4 layers on it. The clustered images had clustered pixels, which improved the performance of the layers in recognizing the objects, contents, and features of the images.
**Algorithm 4** *K*-Means–YOLOv4 Classifier**Require:** Image Dataset
     **Input:** Random Centroid Points
     **Start:** Clustering Pixels
     **while** pixels≠end **do**
           **Select:** Neural Engine Core
           **Assign:** Processing to Core
           **Calculate:** Mean Value
           **Set**: Pixel to Cluster
     **end while**
     **Run:** YOLO’s Backbone on Clustered Image
     **if** Image Contains (COVID) **then**
           **Flag:** Image as Affected
     **else**
           **Flag:** Image as non-Affected
     **end if**
     **Output:** Classified Image


## 7. Performance Evaluation and Datasets

Performance metrics were crucial for evaluating both classification and detection techniques. In addition, the experiment environment, datasets, and data preparation used to assess these metrics were equally important. Therefore, this section provides a detailed explanation of the performance metrics, datasets, and environment, as they related to the obtained results and the implementation of the proposed solution.The dataset consisted of a diverse set of information, which was classified into four distinct categories. The dataset was split into a training set (70%) and a testing set (30%), with the training set being used to train the machine-learning algorithms and the testing set being used to evaluate their performance. The machine-learning algorithms were applied for classification, using features extracted through the feature-engineering process. The proposed algorithm was compared to various categorization approaches and was found to be highly effective on X-ray images in the experiments. The proposed solution was implemented using the Dart ARM-based programming language, which is suitable for resource-constrained mobile devices, along with specialized deep-learning code for machine-learning engines on mobile devices. For iOS devices, the Swift programming language was utilized, which is known for its ease-of-use and safety features, while Kotlin (the native Android language) was employed for Android devices. This approach allowed for the solution to be easily implemented on different mobile devices and platforms, providing a more versatile and widely accessible solution.

The *k*-means-YOLOv4 approach was evaluated on mobile devices equipped with machine-learning engines, including an iPhone 11 Pro Max with a dedicated 16-core machine-learning processor and the Samsung S22 with a system-on-a-chip, featuring a 16-bit floating-point neural processing unit (NPU). The testing dataset was divided into two categories: X-ray images and CT-scan images.

### 7.1. Performance Metrics

Recall (*R*), Precision (*P*), F1-score (*F1*), specificity (*S*), and accuracy were used as the performance criteria to examine deep-learning performance.

- Precision: This metric represented the fraction of genuine positives among the expected positives. As a result, true-positive (*TP*) and false-positive (*FP*) values were important.
  (3) P=TP/(TP+FP)
- Recall: The ratio of true positives accurately categorized by the model was the recall. The recall was calculated using *TP* and *FN* values.
  (4) R=TP/(TP+FN)
- Specificity: This was defined as the proportion of true negatives (those not caused by illness) correctly classified by the model. The *TN* and *FP* values were used to calculate specificity.
  (5) S=TN/(TN+FP)
- F1-Score: The F1-score measured the model’s accuracy by combining precision and recall. Doubling the ratio of the total accuracy and recall values defined the F1-scores.
  (6) F1=2×(P×R)/(P+R)
- Performance (Speed): This was an important performance metric in image detection and data classification and clustering, particularly when dealing with large datasets and real-time applications. It measured the time required to process and analyze the data and produce the desired output. In image detection, speed is important for applications such as autonomous vehicles, surveillance systems, and medical imaging, where the detection and analysis of images must be performed in real-time. The speed metric is usually measured in frames-per-second (FPS), which represents the number of images that can be processed in one second. In data classification and clustering, speed is important for applications such as recommendation systems, fraud detection, and customer segmentation, where large amounts of data must be analyzed and classified in a timely manner. The speed metric is usually measured in terms of processing time or throughput, which represents the number of data points that can be processed per unit of time.

### 7.2. Dataset

To validate the proposed solution in various scenarios and on varied dataset properties, experiments were conducted using a number of different datasets. The characteristics of all the datasets are summarized in Table 1. All datasets were downloaded from the Kaggle website.

A range of dataset sizes was used in this paper to evaluate the performance of the proposed solution with different dataset sizes ranging from a few thousand rows to millions of rows. Therefore, a dataset with 54 MB was used.

For image detection and to confirm the model’s robustness, two independent datasets were collected and tested. The dataset used in this paper was created using the analysis conducted by [60] and can be downloaded at https://github.com/muhammedtalo/COVID-19 (accessed on 20 February 2023). The dataset consisted of 500 pneumonia, 125 COVID-19, and 500 no-findings X-ray images. It was created using two separate resources: X-ray images obtained from multiple open-access sources of COVID-19 patients in the Cohen [61] database, and the chest X-ray database for normal and pneumonia X-ray images, provided by Wang et al. [62]. The COVID-19 dataset included 43 female patients and 82 male patients. Metadata were not available for all patients in this dataset. Positive COVID-19 patients were, on average, around 55 years old. This was a versatile dataset that could be used for multi-class and binary classification tasks.

The dataset from Harvard Lab [55] was also used in this study. The dataset consisted of non-enhanced chest CT scans of more than 1000 individuals diagnosed with COVID-19. The average age of the CT-scan patients was 47.18 years, with a standard deviation of 16.32 years and a range from 6 to 89 years. The population was composed of 60.9% males and 39.1% females. The most common self-reported co-morbidities among patients were coronary artery or hypertension disease, interstitial pneumonia or emphysema, and diabetes. The positive PTPCR patient images were obtained from in-patient treatment sites for COVID-19 and accompanying clinical symptoms, between March 2020 and January 2021. The scans were taken during end-inspiration with the subjects in a supine position.

The CT scans were conducted using a 16-slice helical mode on NeuViz equipment, without the use of intravenous contrast. The images were captured in DICOM format and were 16-bit gray-scale with 512 × 512 px. The slice thickness was determined by the operator and ranged from 1.5 to 3 mm, based on the clinical examination requirements. The CT scans were reviewed for the presence of COVID-19 infection by two board-certified radiologists. In cases where the first two radiologists were unable to reach a consensus, a third more-experienced radiologist provided the final judgment. The CT images showed a variety of patterns indicative of COVID-19-specific lung infections.

In the third phase of our comparison, two datasets were used. The specifics of the two major subsections of the sourced image graphs were as follows.

1. Radiography database for COVID-19 in [63]. The authors gathered chest X-ray images of COVID-19-positive individuals, along with healthy people and those with viral pneumonia, and made them accessible to the public on https://www.kaggle.com/ (accessed on 20 February 2023).
2. Actualmed, Pau Agust Ballester, and Jose Antonio Heredia from Universitat Jaume I (UJI) created the Actualmed COVID-19 Chest X-ray Dataset for study (https://github.com/agchung/Figure1-COVID-chestxray-dataset/tree/master/image (accessed on 20 February 2023)).

A total of 3106 images were utilized for model training, 16% of which were used for model validation. A total of 806 non-augmented images from various categories were used to test the proposed solution and assess the performance.

Furthermore, the large image datasets in Table 2 were used for the big-data evaluation. All the datasets were downloaded from the Kaggle website.

#### Data Preparation

The data clustering had to be prepared, and the primary parameters had to be selected before clustering, as follows:

- **Noise Removal:** The advanced parallel *k*-means clustering algorithm utilized the mean imputation as the method for handling missing data. In this approach, missing values were replaced with the mean value of the corresponding feature across all samples. This method is simple and computationally efficient, and it has been shown to be effective in practice. However, the mean imputation may introduce bias in the clustering results if the missing data were not missing completely-at-random (MCAR). If the missing data were missing-at-random (MAR) or missing not-at-random (MNAR), more sophisticated methods such as regression imputation and multiple imputation could be required to avoid bias.
- **Number of Clusters:** Selecting the optimal number of clusters in the advanced parallel *k*-means clustering was crucial for achieving effective cluster analysis. This is particularly true in the medical field, where the identification of meaningful clusters can lead to more accurate diagnoses and treatments. However, the traditional methods of finding *k*-value, such as the Elbow method or the Silhouette method, are not always sufficient in the medical field, where the data are often complex and high-dimensional. In such cases, expert knowledge could be required to identify clinically relevant subgroups, which could then be used to determine the optimal number of clusters. In this paper, the *k*-value set to 2 in the clustering of numeric and text data and set to 5 for image clustering, as there were 5 main gray-scale stages of colors in the X-ray and MRI images.

### 7.3. Operating System Implementation

Dispatch queues are a feature of the Grand Central Dispatch (GCD) system, which is a part of the iOS and macOS operating systems. GCD provides a high-level, asynchronous programming interface for managing concurrent tasks. Dispatch queues are lightweight and provide a simple interface for executing tasks concurrently without consuming an excessive amount of system resources. Dispatch queues are managed by the operating system and can be used to process tasks on a first-in, first-out (FIFO) basis. This makes it easy to manage task dependencies and avoid competitive conditions, and tasks submitted to a dispatch queue can be executed in parallel with other tasks in the queue. Dispatch queues can be created with different priorities to manage the order of execution of tasks and ensure that high-priority tasks are executed first.

Threads, in contrast, are a lower-level mechanism for achieving concurrency in a program. Threads achieve true parallelism, as multiple threads can execute simultaneously on different processor cores. Each thread has its own stack and program counter, and threads can share memory with other threads in the same process. Threads are managed by the operating system and can be used to process tasks concurrently in a more fine-grained way than dispatch queues. As compared to dispatch queues, threads have a higher overhead and require more system resources, making them less suitable for lightweight tasks. Threads can be used to implement more complex concurrency patterns, such as locking, synchronization, and message-passing.

The proposed solution for implementing the modified parallel *k*-means clustering algorithm on iOS leveraged the advantages of dispatch queues to achieve concurrency. The GCD framework provided several types of queues, including serial and concurrent dispatch queues. A serial dispatch queue executed tasks one at a time, while a concurrent dispatch queue executed tasks concurrently.

In the proposed solution, a concurrent dispatch queue was used to execute the *k*-means clustering algorithm on multiple cores simultaneously. Each task was scheduled on the dispatch queue, and the queue handled the scheduling of tasks across multiple cores. This allowed the algorithm to take advantage of the multi-core neural engine processor and general-purpose processor, leading to improved performance.

Furthermore, GCD provided mechanisms to ensure thread safety and avoid competitive conditions through the use of synchronization techniques, such as semaphores and barriers. By utilizing these features, the implementation of the parallel *k*-means clustering algorithm on dispatch queues was more efficient and reliable.

## 8. Results and Discussion

The proposed work was subjected to thorough testing and evaluation in multiple stages to ensure its effectiveness at various levels and within different contexts. The primary focus was on enhancing performance and leveraging the high speeds offered by the two integrated algorithms.

### 8.1. Operating System Performance

The proposed solution was designed to be operating system independent and hardware accelerated. This meant that the advanced parallel *k*-means clustering could be executed on any operating system that had two processors: a neural engine processor and a general-purpose processor. However, both iOS and Android operating systems were designed to manage and take advantage of hardware allocation and management that included their neural engine processor. These dedicated operating systems were able to send specific tasks to a particular processor core, enabling the implementation and execution of the advanced parallel *k*-means clustering.

Overall, this type hardware acceleration provides opportunities for future advancements of the operating systems, which is expected since the new M-family MacOS already supports dedicated neural-engine-core assignments.

In order to evaluate the performance of the advanced *k*-means clustering across different operating systems, Table 3 presents two large datasets, each with over 9 million records. These were clustered using the advanced parallel *k*-means clustering algorithm on Windows OS, Android, and iOS systems.

The performance results, as presented in Table 4, showed that the processing of 11 million records from the Google Play Store dataset doubled in speed with a dedicated ML processor. The next experiment was conducted using the education-sector dataset, and the mobile processor exhibited a performance up to 10-times faster than the desktop OS (Windows 11). Additionally, the performance of iOS was twice as fast as that of the Android OS.

The performance differences observed between the iOS and Android operating systems, within the context of advanced parallel *k*-means clustering, could be due to several factors. It could be related to the differences in the underlying architectures of the two operating systems. Specifically, iOS was designed to take full advantage of its hardware resources, including the dedicated neural engine cores, which could explain the observed faster performance, as compared to Android.

Additionally, the iOS architecture was based on the use of Objective-C and dispatch queues, which were designed to facilitate concurrent processing and task scheduling. These features provide a more efficient way to execute the parallel *k*-means clustering algorithm, potentially resulting in the observed faster performance.

However, the performance differences observed could have also been influenced by other factors, such as the differences in the hardware configurations of the devices used to test the algorithms, as well as the specific implementation of the parallel *k*-means clustering algorithm on the different operating systems.

### 8.2. Data Classification Model

The performance of logistic regression and naive Bayes algorithms could have been influenced by various factors, such as the size and complexity of the data, the hardware and software utilized, and the specific implementation of the algorithms. Typically, logistic regression has been faster than naive Bayes when working with large datasets, as the naive Bayes algorithm can become computationally demanding as the number of features increases. However, naive Bayes can be faster when working with smaller datasets or when the number of features is relatively limited. Table 5 illustrates the performance of both algorithms after classifying 10 million records of medical data. The table shows the speed and accuracy of both algorithms, which aided in determining which algorithm was more suitable for a specific application. While the speed of an algorithm was an important consideration, accuracy was also taken into account when a compromise between speed and accuracy may be necessary.

Based on the datasets described in Section 7.2, the proposed solution was analyzed to evaluate its classification performance and accuracy.

The results presented in Table 6 and Table 7 demonstrated the superior performance of the proposed solution, as compared to the logistic-regression and naive Bayes algorithms. The naive Bayes algorithm is known to be efficient for small datasets, but the proposed solution outperformed both algorithms, even when the dataset size increased. This highlighted the effectiveness of the proposed solution in handling larger datasets, which pose a significant challenge for traditional classification methods. Additionally, the strong performance of the proposed solution, as compared to the standard classification algorithms, such as logistic regression and naive Bayes, further emphasized its potential for practical applications. Overall, the results demonstrated the exceptional performance and potential of the proposed solution.

One possible reason for the higher accuracy of the proposed solution was that it had been specifically designed to handle larger datasets, which may have been more challenging for traditional classification algorithms. For example, logistic regression and naive Bayes algorithms could have struggled to effectively classify data when the number of features increased significantly, as they can become computationally demanding as the number of features increases. In contrast, the proposed solution used more advanced techniques, including the advanced parallel *k*-means, parallel logistic regression, and the neural engine processor, to effectively classify the large datasets. Additionally, the proposed solution incorporated additional factors and features that were relevant to the classification task, which further improved its accuracy. Overall, the results demonstrated the effectiveness of the proposed solution in handling large datasets and achieving high accuracy in classification tasks.

In order to examine the performance of the proposed solution with the recent advancements in medical data classification, the proposed solution was compared with the three most recent medical-data-classification approaches, which were: [65,66,67]. All solutions were compared with the proposed solution in terms of classification performance and classification accuracy, as shown in tables below.

As shown in Table 8, the proposed solution significantly outperformed the three compared solutions, while the naive Bayes-based algorithm tended to be slower, the proposed solution was more effective than both the binary logistic regression and the logistic regression. This suggested that the proposed solution was particularly well suited for handling larger datasets, which could be more challenging for traditional classification algorithms. The results demonstrated the strong performance of the proposed solution, as compared to the conventional classification algorithms, indicating that it was an effective and reliable method for classification tasks.

The accuracy of the proposed solution was compared with previous solutions, and the results, as shown in Table 9, demonstrated its high accuracy. Specifically, the proposed solution outperformed the comparable solutions, achieving an accuracy rate of 99.8% while a novel binary-logistic-regression solution only achieved 98% accuracy. The worst performance in terms of accuracy was observed in the logistic-regression solution designed for the prediction of myocardial infarction disease in [66]. These results suggested that the proposed solution was particularly effective at achieving high accuracy in classification tasks, and that it outperformed other approaches.

### 8.3. Training Proposed Model

**Figure 6:**
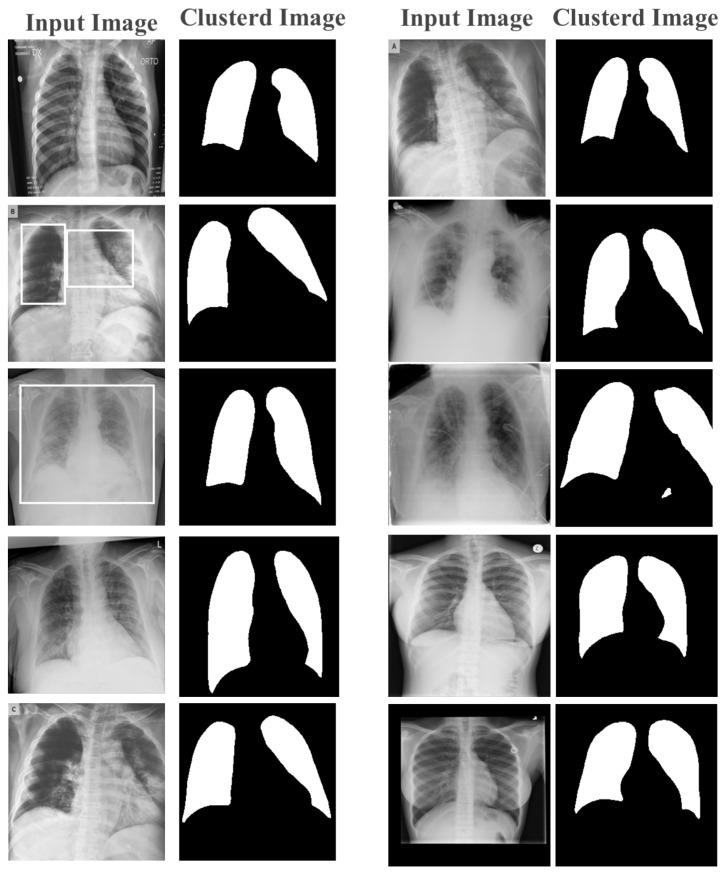
Learned features from the first layer.

Feature extraction and classification are the two crucial components of the proposed image detection system. The quality of the extracted features was critical to the success of the classification process. Therefore, the extracted features were used to train the model in order to demonstrate its effectiveness in feature extraction. Figure 6 shows an image after applying the *k*-means clustering technique with feature selection. The resulting image was divided into two main clusters, black and white, in the first stage of learning. This clustered image was then used as a map for pixel-based feature extraction, where each pixel was assigned to its corresponding cluster based on the mapped image.

In the next step, the pixel values were processed with their original values for the image detection process. This approach provided two benefits. Firstly, any outlier pixels due to the X-ray device or CT-scan process were removed. Secondly, a new feature was added to the image pixels, which was the pixel group. The cluster value associated with each pixel provided valuable information for image feature extraction and detection. By considering the cluster value, we could efficiently extract the relevant features from the image and ignore the noise and other irrelevant pixels. This approach significantly improved the accuracy of image detection and reduced false positives. When processing an X-ray image, the proposed solution began by extracting the lung features of the patient and then determined whether the lungs were normal or abnormal by classifying them as positive or negative, accordingly.

### 8.4. Object Detection Speed

During the initial phase, the proposed work was compared with a range of standard algorithms frequently used for image classification. The proposed solution demonstrated exceptional performance, outperforming the other algorithms by up to 15-fold. It also outperformed the YOLOv4 algorithm by approximately 60%, as shown in the comparison presented in Table 10.

To assess the performance of the proposed solution under various scenarios and with varied device specifications, it was tested using both a standard computer CPU (Intel Core i5-3.5 GHz) and GPU (AMD Radeon R9 M290X 2 GB). The results of the experiments showed that the proposed detection solution maintained its high performance, as compared to the YOLOv4 algorithm, as demonstrated in Table 11.

The results showed that the proposed solution exhibited a high performance, which was up to 2.3 times faster on the GPU and up to 1.5 times faster on the CPU, as compared to the standard YOLOv4. Additionally, the proposed algorithm demonstrated a significant speed advantage, achieving speeds that were up to 7 times faster due to the high speed of the proposed algorithm and the efficient use of the artificial intelligence processors in modern mobile devices, as compared to recent solutions, such as (VGGCOV19-NET [70] and CAD-based YOLOv4 [71]).

### 8.5. Object Detection Performance

In the second phase of the performance comparison, as shown in Table 12, the proposed solution was compared with two recent approaches that made adjustments to classification algorithms to handle X-ray images of COVID-19 patients.

**Figure 7:**
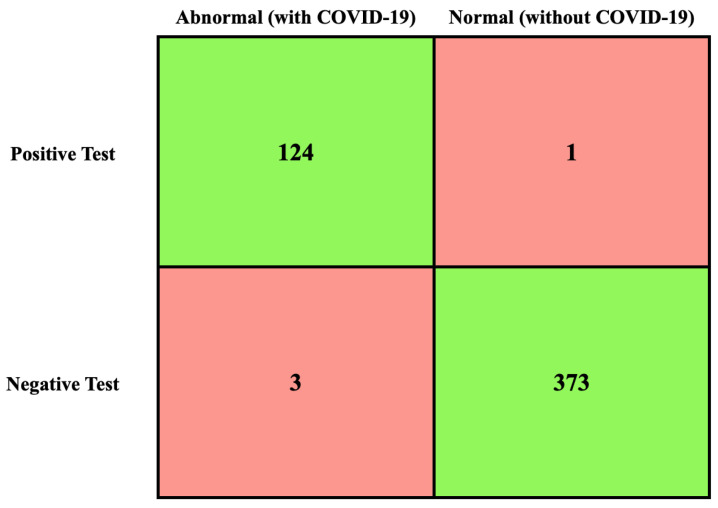
*TN*, *TP*, *FN*, and *FP* values of X-ray dataset.

Due to the importance of the TN, TP, FN, and FP values [72], their values had been calculated first, as shown in Figure 7.

In the last part of the comparison, the proposed work was compared to the benchmark examples, based on four performance measures, including recall, precision, F1-score, and accuracy. These represented the best testing factors for evaluating the performance of the classification algorithms and to ensure that the improvements achieved [73] by the proposed algorithm were accurate across all levels, which, in turn, would indicate its potential application in the medical field. The results, as shown in Table 13, illustrated the excellent performance of the proposed algorithm in the classification task of images when applied to the Fold 1–5 levels.

When the algorithm treated images classified as infected images, it also showed superior accuracy, and the performance measures of the rest of the results are shown in Table 14 and Table 15.

An advanced *K*-means clustering [6] combined with YOLOv4 solution enabled the rapid and accurate detection of COVID-19 within milliseconds, making it a useful tool in regions with a shortage of experienced doctors and radiologists. Additionally, the model could be utilized to identify patients in settings with limited healthcare facilities, even when only X-ray technology is available, and it could ensure more timely treatments for positive COVID-19 patients. One practical benefit of the concept was that it allowed for the identification of patients who did not require PCR testing, thereby reducing the overcrowding in medical facilities.

In the second part of the performance comparison, as shown in Table 16, the proposed solution was compared with recent studies in which classification algorithms were modified to handle CT-scan images of COVID-19 patients. Due to the high speed of the suggested method and the extensive use of artificial intelligence processors prevalent in recent mobile devices, the proposed algorithm demonstrated superiority in its accuracy, recall, and other performance metrics.

**Figure 8:**
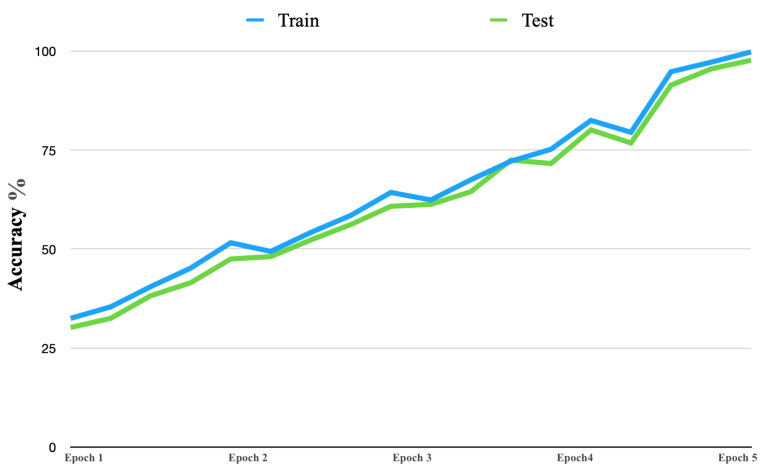
Accuracy learning curve.

Figure 8 shows the learning curve accuracy of the proposed solution in both the training and testing stages. The accuracy of the proposed solution had consistent improvement. Furthermore, the learning curve began with an accuracy near 32% and continued to improve, up to 99%.

In the third part of the performance comparison, as shown in Table 17, the proposed solution was compared with recent studies that used a classification technique on brain MRI images to maximize the generalizability of the proposed solution. This comparison was conducted to show that the proposed solution could be adapted for various datasets and image types, as well as to classify other diseases, such as brain tumors. The results showed that the proposed solution had excellent performance across all four comparison parameters (recall, precision, F1-score, and accuracy). The dataset used in [75], which consisted of 280 samples of MRI images, was also used in this test. The dataset contained 100 images with normal tumors and 180 with abnormal tumors.

Table 17 shows the proposed solution’s performance, as compared to a recent solution [75]. The results showed that the proposed solution outperformed the comparable solution, with an accuracy of up to 98%.

The proposed solution was compared with a high-performance and highly accurate solution, which had been proposed in 2020 [76]. For datasets 1 and 2, the solution obtained 98.7%, 98.2%, 99.6%, and 99% for classification accuracy and F1-Score, respectively. However, as shown in Table 18 and Table 19 with the best values across 7 folds, the proposed solution had excellent classification performance in terms of accuracy, recall, precision, and F1-score, as compared to the comparable 2020 approach.

The excellent performance and accuracy of the proposed solution could be attributed to the optimization of the *k*-means clustering, which enhanced the recognition of the image characteristics by the classifier. Additionally, the optimization of the YOLOv4 algorithm through modified layers improved the ability to detect and recognize features, resulting in an overall improvement in performance.

In order to evaluate the performance of the proposed solution on a vast amount of medical image detection, a set of big-medical-data was used, as described in Section 7.2 and (Table 3). Table 20 shows the performance of the proposed solution, as compared to recent approaches. The performance of the proposed solution in terms of recall, precision, F1-score and accuracy was up to 10% better than the comparable solutions.

The results of the proposed approach using advanced parallel *k*-means clustering, logistic regression, and YOLOv4 for medical data classification and image detection could have important implications for the field of healthcare. The accurate classification and detection of medical data could have a significant impact on patient outcomes by enabling earlier diagnoses and more effective treatment planning. The proposed approach has potential for improving the accuracy and efficiency of these tasks, which could ultimately lead to better patient outcomes and reduced healthcare costs.

Furthermore, the proposed approach has the potential to contribute to the development of new solutions in these areas by providing a more efficient and effective means of pre-processing medical data. The use of advanced parallel *k*-means clustering for pre-processing reduced the dimensionality of the data, which made it easier to classify and detect patterns. This could lead to the development of new algorithms that are more effective for identifying specific medical conditions and abnormalities and could, ultimately, lead to new treatments and therapies.

Additionally, the proposed approach could aid in the development of new medical imaging technologies. By improving the accuracy of image detection, the proposed approach could assist in identifying abnormalities that are difficult to detect using traditional imaging methods. This could lead to the development of new imaging technologies that are more accurate and effective and could, ultimately, improve patient outcomes.

In terms of the overall medical-data field, the proposed approach using advanced parallel *k*-means clustering for pre-processing medical data, combined with logistic regression and YOLOv4 for classification and image detection, respectively, could contribute to the development of new solutions for medical data classification and image detection.

Firstly, the use of advanced parallel *k*-means clustering for pre-processing medical data could significantly reduce the processing time and improve the accuracy of subsequent classification and detection tasks. This could be especially beneficial for large-scale medical datasets, where traditional clustering methods may not be feasible due to computational limitations.

Secondly, the combination of logistic regression and YOLOv4 for classification and image detection, respectively, could improve the accuracy of these tasks in medical applications. Logistic regression is a simple and efficient algorithm that could be used for both binary and multi-class classification, while YOLOv4 is a state-of-the-art object detection algorithm that can detect multiple objects in an image with high accuracy.

Thirdly, the proposed approach could potentially aid in the diagnosis, treatment planning, and disease monitoring in healthcare. The accurate classification and detection of medical data could provide clinicians with valuable insights into a patient’s condition and assist them in making informed decisions regarding treatments.

Lastly, the proposed approach could also serve as a framework for the development of new solutions in medical data classification and image detection. The combination of advanced clustering methods, logistic regression, and object detection algorithms could be customized and optimized for specific medical applications and datasets. This could lead to the development of innovative solutions that address the unique challenges and complexities of medical data analysis.

## 9. Conclusions

The proposed approach using advanced parallel *k*-means clustering for pre-processing medical data, combined with logistic regression and YOLOv4 for classification and image detection, respectively, effectively improved the performance of these algorithms, particularly when applied to large medical datasets. The results of the classification task showed that the approach was able to accurately classify the medical data, and the results of the image detection task using X-ray and CT scan images showed that the approach was able to effectively detect and classify the medical images. The use of advanced parallel *k*-means pre-processing and acceleration of the neural engine processor contributed to the improved accuracy and efficiency of the approach. This approach has the potential to significantly impact the field of healthcare, as it can aid in diagnostics, treatment planning, and disease monitoring. Further research and evaluation on larger and more diverse medical datasets could reveal additional benefits and potential applications. While the proposed solution has shown promise in improving the accuracy and efficiency of these tasks on large medical datasets, there were still limitations that should be considered. One limitation was the hardware dependency, as the acceleration of the *k*-means clustering was highly dependent on the neural engine processor, multi-core processor, and the operating system’s support for hardware management. Another limitation was the ability to improve 24-bit color images, which require a different number of *k*-values and could affect the clustering performance negatively.

## Figures and Tables

**Table 1:** Clustering datasets.

Dataset	Dataset Size
COVID-19 Dataset	54 MB
COVID-19	362 MB
COVID-19 Open Research Dataset Challenge	20 GB

**Table 2:** Clustering datasets.

Dataset	Dataset Size	No. of Images/Slices	No. of Classes
Large COVID-19 CT-scan-slice dataset	2 GB	7593	9
COVIDx CT	65 GB	194,922	10
CT Low-Dose Reconstruction	20 GB	16,926	6

**Table 3:** Clustering datasets.

Dataset	Number of Records
Google Play Store	11,000,000
KDD99 [64]	9,000,000

**Table 4:** Clustering performance of big-data sets in minutes.

Dataset	Windows OS	iOS	Android OS
Google Play Store	90 min	46.1	56.4
Education Sector	24.3 ms	2.4	6.3

**Table 5:** Data classification performance.

Algorithm	Performance (m.)
Logistic Regression	23.1
Naive Bayes	31.4

**Table 6:** Data classification performance (Dataset 1).

Dataset	Algorithm	Speed (m.)
1	Logistic Regression	12.2
	Naive Bayes	8.5
	*K*-Means–Logistic Regression	9.7
2	Logistic Regression	63.2
	Naive Bayes	83.1
	*K*-Means–Logistic Regression	45.1
3	Logistic Regression	2754
	Naive Bayes	3571
	*K*-Means–Logistic Regression	1693

**Table 7:** Data classification accuracy of Dataset 1.

Dataset	Algorithm	Accuracy (%)
1	Logistic Regression	93.4
	Naive Bayes	92.1
	*K*-Means–Logistic Regression	95.3
2	Logistic Regression	94.2
	Naive Bayes	93.5
	*K*-Means–Logistic Regression	97.2
3	Logistic Regression	93.1
	Naive Bayes	91.3
	*K*-Means–Logistic Regression	97.6

**Table 8:** Data classification speed (min.) when compared with recent approaches.

Algorithm	Performance (m.)
Logistic Regression [66]	34.1
Novel Binary Logistic Regression [65]	28.3
Correlated Naive Bayes [67]	37.4
*K*-Means–Logistic Regression	21.1

**Table 9:** Data classification accuracy when compared with recent approaches.

Algorithm	Accuracy (%)
Logistic Regression [66]	88
Novel Binary Logistic Regression [65]	98
Correlated Naive Bayes [67]	97
Shared Bayesian Variable Shrinkage [68]	93
Classification of Breast Cancer Metastasis Using Machine-Learning Algorithms [69]	92
*K*-Means–Logistic Regression	99.8

**Table 10:** Detection speeds of object algorithms.

Algorithm	Speed (ms.)
SPP-net	1500
R-CNN	900
Fast R-CNN	750
Faster R-CNN	600
R-FCN	550
Mask R-CNN	400
YOLOv3	250
YOLOv4	150
Advanced Parallel *K*-means–YOLOv4 (APK-YOLO)	90

**Table 11:** Classification performance on neural engine, CPU, and GPU.

Algorithm	CPU (ms.)	GPU (ms.)	Neural Engine (ms.)
YOLOv4	350	280	150
APK-YOLOv4	220	120	90

**Table 12:** Image classification performance comparison.

Algorithm	Speed (ms.)
VGGCOV19-NET [70]	620
CAD-based YOLOv4 [71]	530
APK-YOLOv4	90

**Table 13:** Recall, precision, F1-score, and accuracy performance of Folds 1–5 of the chest X-ray images.

Fold	Algorithm	Recall	Precision	F1	Accuracy
1	VGGCOV 19-NET [70]	78.20	78.80	78.30	78.22
	CAD-based YOLOv4 [71]	75.90	75.6	75.8	75.4
	APK-YOLOv4	82.2	82.6	82.7	82.7
2	VGGCOV 19-NET [70]	91.10	91.10	91.10	91.11
	CAD-based YOLOv4 [71]	89.5	89.4	89.5	89.5
	APK-YOLOv4	93.4	93.4	93.3	93.4
3	VGGCOV 19-NET [70]	84.40	84.80	84.50	84.44
	CAD-based YOLOv4 [71]	90.2	90.1	90.2	90.1
	APK-YOLOv4	94.2	94.23	94.2	94.3
4	VGGCOV 19-NET [70]	95.10	95.20	95.10	95.11
	CAD-based YOLOv4 [71]	94.5	94.2	94.4	94.4
	APK-YOLOv4	96.7	96.4	96.5	96.5
5	VGGCOV 19-NET [70]	95.60	95.70	95.60	95.56
	CAD-based YOLOv4 [71]	94.2	94.1	94.2	94.2
	APK-YOLOv4	97.2	97.6	97.4	97.5

**Table 14:** Recall, precision, F1-score, and accuracy performance on COVID-19 images.

Algorithm	Recall	Precision	F1	Accuracy
VGGCOV 19-NET [70]	92.80	99.15	95.87	87.89
CAD-based YOLOv4 [71]	91.5	95.7	85.7	90.67
APK-YOLOv4	93.8	99.7	97.44	96.21

**Table 15:** Recall, precision, F1-score, and accuracy performance on no-findings images.

Algorithm	Recall	Precision	F1	Accuracy
VGGCOV 19-NET [70]	90.20	86.40	88.26	85.80
CAD-based YOLOv4 [71]	89.1	82.3	80.4	89.7
APK-YOLOv4	92.9	95.4	92.6	91.82

**Table 16:** Recall, precision, F1-score, and accuracy performance of Folds (1–5) with CT-scan images.

Fold	Algorithm	Recall	Precision	F1	Accuracy
1	Compressed Chest CT Image through Deep Learning [74]	79.10	79.30	79.10	79.87
	APK-YOLOv4	83.5	83.4	83.4	83.3
2	Compressed Chest CT Image through Deep Learning [74]	93.10	92.80	92.60	92.8
	APK-YOLOv4	94.1	94.3	94.7	94.6
3	Compressed Chest CT Image through Deep Learning [74]	89.10	89.70	89.60	89.8
	APK-YOLOv4	93.8	94.1	93.9	93.8
4	Compressed Chest CT Image through Deep Learning [74]	96.20	96.30	96.18	96.20
	APK-YOLOv4	97.1	97.5	97.4	97.2
5	Compressed Chest CT Image through Deep Learning [74]	98.78	98.75	98.80	98.7
	APK-YOLOv4	99.4	99.7	99.3	99.2

**Table 17:** Recall, precision, F1-score, and accuracy performance of Folds (1–5) on MRI images.

Fold	Algorithm	Recall	Precision	F1	Accuracy
1	hybrid deep CNN-Cov-19-Res-Net [75]	78.50	78.70	78.40	78.12
	APK-YOLOv4	82.4	82.3	82.4	82.3
2	hybrid deep CNN-Cov-19-Res-Net [75]	91.30	91.20	91.60	91.3
	APK-YOLOv4	93.5	93.4	93.2	93.3
3	hybrid deep CNN-Cov-19-Res-Net [75]	90.10	90.2	90.3	90.1
	APK-YOLOv4	94.5	94.5	94.3	94.3
4	hybrid deep CNN-Cov-19-Res-Net [75]	95.30	95.20	95.10	95.40
	APK-YOLOv4	96.3	96.5	96.3	96.2
5	hybrid deep CNN-Cov-19-Res-Net [75]	97.18	97.15	97.30	97.2
	APK-YOLOv4	98.1	98.2	98.1	98.1

**Table 18:** Data classification (recall, precision, accuracy, and F1-score) on dataset 1.

Algorithm	Recall	Precision	F1	Accuracy
Deep Features and Fractional-Order Marine Predators [76]	98.2	98.5	99.6	98.7
APK-YOLOv4	98.8	99.1	99.8	99.1

**Table 19:** Data classification (recall, precision, accuracy, and F1-score) on dataset 2.

Algorithm	Recall	Precision	F1	Accuracy
Deep Features and Fractional-Order Marine Predators [76]	97.7	98.1	99	98.2
APK-YOLOv4	98.5	99.3	98.1	99.6

**Table 20:** Image detection recall, precision, F1-score, and accuracy performance of Folds (1–5) (big-medical-data image sets).

Fold	Algorithm	Recall	Precision	F1	Accuracy
1	VGGCOV 19-NET [70]	77.30	77.20	77.10	77.34
	CAD-based YOLOv4 [71]	78.80	78.9	78.5	78.4
	APK-YOLOv4	85.1	84.1	85.3	85.7
2	VGGCOV 19-NET [70]	90.30	90.40	90.60	90.4
	CAD-based YOLOv4 [71]	90.6	91.2	91.6	91.8
	APK-YOLOv4	94.3	95.1	95.2	95.1
3	VGGCOV 19-NET [70]	88.60	88.85	87.40	87.74
	CAD-based YOLOv4 [71]	91.3	91.1	91.5	91.4
	APK-YOLOv4	96.52	96.27	97.1	96.8
4	VGGCOV 19-NET [70]	95.30	95.24	95.34	95.61
	CAD-based YOLOv4 [71]	94.7	94.25	94.7	94.8
	APK-YOLOv4	97.7	98.5	97.8	97.9
5	VGGCOV 19-NET [70]	96.60	96.30	95.9	95.76
	CAD-based YOLOv4 [71]	95.2	95.13	95.22	95.12
	APK-YOLOv4	98.8	98.4	98.7	98.6

## Data Availability

COVID-19 Research Challenge: (https://www.kaggle.com/datasets/allen-institute-for-ai/CORD-19-research-challenge); Large COVID- 19 CT (https://www.kaggle.com/datasets/maedemaftouni/large-covid19-ct-slice-dataset); COVIDx- CT (https://www.kaggle.com/datasets/hgunraj/covidxct); CT- LOW- Dose (https://www.kaggle.com/datasets/andrewmvd/ct-low-dose-reconstruction); Google Play Store (https://www.kaggle.com/datasets/gauthamp10/google-playstore-apps); KDD99 (https://datahub.io/machine-learning/kddcup99).
